# Hollow Silica Cubes with Customizable Porosity

**DOI:** 10.3390/ma13112474

**Published:** 2020-05-29

**Authors:** Samuel Hugh Gallagher, Olivier Trussardi, Oliver Lipp, Dominik Brühwiler

**Affiliations:** Institute of Chemistry and Biotechnology, Zurich University of Applied Sciences (ZHAW), CH-8820 Wädenswil, Switzerland; samuel.gallagher@zhaw.ch (S.H.G.); otrussardi@hotmail.com (O.T.); oliver.lipp@zhaw.ch (O.L.)

**Keywords:** mesopores, hollow particles, silica, monolayers, pseudomorphic transformation

## Abstract

Hollow silica cubes were synthesized by a deposition of a thin silica shell onto micrometer-sized hematite cubes. Ordered mesopores with well-defined pore diameters of 2.8 nm and 3.8 nm were introduced into the silica shell by means of pseudomorphic transformation after removal of the hematite core. The particles retained their cubic morphology upon pseudomorphic transformation, allowing for the preparation of close-packed layers of the hollow mesoporous silica cubes by drop-casting and the visualization of the hollow core by focused ion beam scanning electron microscopy.

## 1. Introduction

Ordered mesoporous materials contain advantageous properties for numerous applications, as a result of their large specific surface areas, defined pore structures and variable surface chemistry. These properties are already used to great effect in the fields of drug delivery [1,2,3,4], catalysis [5,6,7,8], heavy metal adsorption [9,10,11] and sensing [12,13,14], as well as model systems for pore condensation studies [15,16,17,18,19]. The void volume available for the adsorption of guest species can be maximized by the use of hollow particles. A common synthetic approach towards well-defined hollow silica particles is to condense silica precursors in the presence of a hard template (acting as a core), followed by the removal of the core under conditions which leave the silica shell intact [20,21]. This method results in the template dictating the final morphology of the silica shell [22,23,24]. A number of template morphologies and sizes have been utilized, but seldom presented are cubic morphologies, although this shape offers attractive options for dense packing [25].

The typical pathway of silica synthesis via the hydrolysis and the condensation of an alkoxysilane yields an amorphous macroporous silica shell upon the removal of the core. Despite the favorable increase in accessible void volume, the silica shell does not feature defined pores, and can therefore not be utilized to establish gating systems for the stimuli-responsive releases of the guests [2]. The introduction of an ordered mesoporous framework into a macroporous material can be achieved by pseudomorphic transformation (PT) [26,27,28,29,30]. The process of PT is based on the careful control of dissolution and reprecipitation of silica in the presence of a structure-directing agent (SDA) such as hexadecyltrimethylammonium bromide (CTAB). The product of a PT with CTAB displays a similar structure to that of an MCM-41 type material [31], although it has been observed that the pore structure of the starting material can affect the pore structure (e.g., the pore spacing and connectivity) of the transformed product [32].

The preparation of hollow mesoporous silica cubes (HMSCs) was achieved through the synthesis of hollow silica cubes by templating with cubic hematite crystals [33], and subsequent PT with alkyltrimethylammonium SDAs. PT can thereby in principle be conducted before or after the extraction of the hematite core. Assembly of the HMSCs into close-packed layers allowed for the convenient investigation of the shell thickness and particle integrity by means of focused ion beam milling, combined with scanning electron microscopy (FIB-SEM).

## 2. Materials and Methods

### 2.1. Chemicals

Iron (III) chloride hexahydrate (FeCl_3_·6H_2_O, ACS reagent, 97%), NaOH (reagent grade, ≥98%, pellets), polyvinylpyrrolidone (PVP, 40,000 g mol^−1^), ethanol (96%), tetramethylammonium hydroxide solution (TMAOH, 10% in H_2_O), tetraethyl orthosilicate (TEOS, ≥98%), hydrochloric acid (ACS reagent, 37%), hexadecyltrimethylammonium bromide (CTAB, ≥98%) and dodecyltrimethylammonium bromide (DTAB, ≥98.0% (NT)) were purchased from Sigma–Aldrich (Sigma-Aldrich Chemie GmbH, Buchs, Switzerland) and used without further purification.

### 2.2. Synthesis of Pseudo-Cubic Hematite

In a 100 mL Pyrex bottle, FeCl_3_·6H_2_O (27.03 g, 0.1 mol) was dissolved in doubly distilled H_2_O (40 mL) by stirring for 15 min at room temperature. All handling of FeCl_3_·6H_2_O was completed with polypropylene equipment, and washed into the Pyrex bottle with an additional amount of H_2_O (10 mL). The Pyrex bottle was then placed in an oil bath (75 °C), and the solution was stirred at 500 rpm for an additional 15 min. To a separating funnel, an aqueous NaOH solution (6 M, 45 mL, 0.27 mol) was added and positioned over the Pyrex bottle [34]. The NaOH solution was added to the iron chloride solution in under 10 min, with special attention on the final 5 mL as coagulation is prominent. If coagulation occurred, the stirring rate was increased. After complete combination, the mixture was stirred for an additional 5 min at 75 °C. The Pyrex bottle was then sealed and transferred to an oven set to 100 °C, and the mixture was allowed to age for four days. The product was recovered by centrifugation and washed with H_2_O (25 mL). The solution should be clear before a final wash with ethanol (25 mL) is completed. The particles were dried in an oven at 80 °C overnight.

### 2.3. Deposition of the Silica Shell

Hematite cubes (1.00 g) were weighed into a 50 mL centrifuge tube and dispersed in H_2_O (14.3 mL). The resulting suspension was subsequently sonicated for 10 min. In a conical flask, PVP (1.43 g, 0.036 mmol) was dissolved in H_2_O (14.3 mL) by stirring at room temperature. The dispersed hematite cubes were added to the PVP solution, and the resulting suspension was stirred for 18 h. The product was centrifuged (4000 rpm, 10 min) and washed with H_2_O (two times at 25 mL). The mass of the wet product was taken and used to calculate the contents of the following mixture. A suspension containing 7.0 wt% of hematite cubes was made in ethanol (15.8 mL). This was added to a solution containing ethanol (190 mL), H_2_O (20 mL) and a TMAOH solution (3% in H_2_O, 1.7 mL, 0.56 mmol). The combined mixture was agitated with an overhead stirrer for 60 min. An ethanolic solution comprising equal volumes of ethanol and TEOS (4.35 mL each) was then dropped into the suspension containing the PVP-coated hematite. The addition rate of 0.073 mL min^−1^ was controlled by means of a syringe pump. During the addition, the suspension was stirred, sonicated and the temperature of the suspension slightly rose. Immediately after the TEOS addition, a solution containing pre-dissolved PVP (1.59 g, 0.04 mmol) in ethanol (30 mL) was added. Stirring was continued under sonication for 60 min, and the beaker was then transferred to a plate with magnetic stirring. Following overnight stirring, the particles were washed with H_2_O (two times 25 mL) and ethanol (25 mL), and then transferred to an oven to dry at 80 °C.

### 2.4. Extraction of the Hematite Core

To remove the hematite cores, the particles were transferred into a 300 mL conical flask, and hydrochloric acid (6 M, 200 mL) was added. The suspension was magnetically stirred overnight, and the following day a light-yellow solution was present. The colorless hollow silica cubes were obtained by centrifugation, washed with H_2_O and left to dry at 80 °C for 18 h. Successful extraction of the hematite core was confirmed by energy-dispersive X-ray spectroscopy (EDS, Figure S1).

### 2.5. Pseudomorphic Transformation

In a typical synthesis, the dried hollow silica cubes were weighed, and this value was used to calculate the reactant ratios. Typically, the hollow silica cubes (100 mg), CTAB (60.6 mg, 0.166 mmol) (or DTAB (71.7 mg, 0.233 mmol)) and H_2_O (1.0 mL) were mixed and stirred for 30 min. NaOH (9.0 mg for the upper limit of PT, 6.4 mg for the lower limit of PT) was added, and the mixture was stirred for an additional 30 min. The resulting slurry was transferred to a Teflon-lined autoclave, where it was kept at 100 °C for 6 h. The product was filtered and washed with 250 mL of H_2_O. The SDA was removed via calcination (550 °C for 5 h after 12 h of drying at 80 °C and a heating rate of 1 °C min^−1^).

### 2.6. Preparation of Monolayers

HMSCs (0.3 mg) were dispersed in H_2_O (1 mL) in an Eppendorf tube and sonicated for at least 90 min. A volume of 40 µL of the suspension was extracted and deposited evenly onto a clean glass sheet (18 mm^2^). The glass sheet was left to air dry overnight on an even surface.

### 2.7. Physical Measurements

The argon sorption isotherms were measured at 87.3 K with a Quantachrome Autosorb iQ MP equipped with a CryoCooler, while krypton sorption isotherms were measured at 77 K. All samples were vacuum-degassed at 150 °C for 3 h prior to the sorption measurements. The pore size distributions and average pore diameters were determined from the adsorption branch by a non-local density functional theory (NLDFT) model developed for silica exhibiting cylindrical pore geometry (Software ASiQwin v3.01, Quantachrome Instruments, Boynton Beach, FL, USA) [35]. The total pore volume (V_tot_) was calculated from the amount of adsorbed argon at a relative pressure of ca. 0.95. The primary mesopore volume (V_p_) was determined from the contribution of the pores with sizes in the range of 2 to 8 nm (NLDFT, Figure S2). The specific surface area (S_BET_) was calculated using points in the relative pressure range between 0.05 and 0.3 according to the Brunauer–Emmett–Teller (BET) model [36]. The cavity volume of HMSCs was estimated by assuming a silica framework density of 2.2 g cm^−3^, V_tot_ = 0.61 cm^3^ g^−1^, a cube length of 1 µm and a shell thickness of 0.1 µm. The scanning electron microscopy (SEM) images were obtained with a Quanta FEG 250. All samples were gold coated before SEM images were taken. EDS was performed with an AMETEK Octane plus on uncoated samples. The FIB-SEM images were acquired with a Zeiss Auriga 40 CrossBeam. The samples were deposited on a Si wafer, carbon tape and aluminum stubs. A 20 nm-thick layer of Pt was deposited on the sample, and sectioning was completed with a 240 nA beam current. The small-angle X-ray scattering measurements (SAXS) were performed using a XENOCS instrument at ICSM (Institut de Chimie Séparative de Marcoule). The wavelength of the incident X-ray beam was λ = 0.71 Å (Mo-radiation), the sample-detector distance was 750 mm and the sample was in a 2.0 mm glass capillary tube. The measurements were acquired for 600 s, and performed at ambient temperature (25 °C).

## 3. Results and Discussion

### 3.1. General Synthesis Pathway

**Figure 1:**
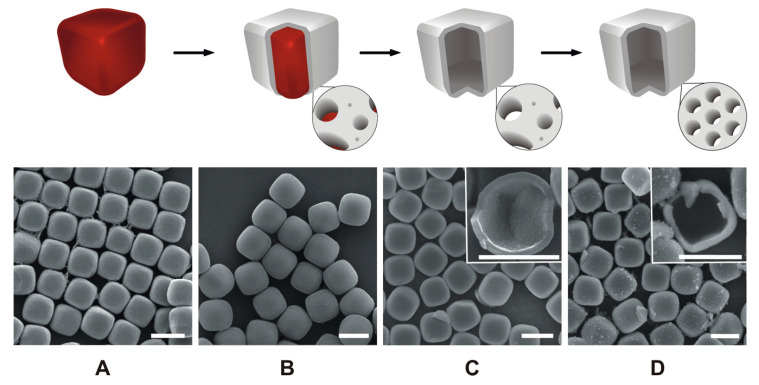
Schematic illustration of the synthesis pathway with corresponding SEM images of the different stages. Hematite cubes (**A**), silica-coated hematite cubes (**B**), hollow silica cubes (**C**) and hollow mesoporous silica cubes (HMSCs) obtained after PT (**D**). The insets of C and D show fractured particles. The scale bars are 1 µm.

The synthesis of HMSCs comprises four steps, as schematically depicted in Figure 1. Hematite cubes are prepared in the first step, and subsequently coated with a thin silica layer by the hydrolysis and condensation of TEOS. The hematite is extracted in the third step. In the final step, defined mesopores are introduced by pseudomorphic transformation (PT). Extraction of the hematite can also be conducted after PT. However, mass transport is strongly limited by the narrow mesopores, leading to a significantly increased duration for complete extraction after PT. The SEM images in Figure 1 illustrate the preservation of the cubic particle morphology throughout the entire process.

### 3.2. Hollow Silica Cubes

Hydrolysis of FeCl_3_ in a 6 M aqueous NaOH solution and subsequent aging at 100 °C led to uniform hematite cubes with a length of approximately 1 µm. To reduce agglomeration between hematite cubes, PVP (40 kg mol^−1^) was added in solution to the hematite cubes. PVP has been reported to improve the formation of silica shells through its physisorption to the surface of the hard template [37,38]. The PVP concentration and chain length must be carefully chosen to match the particle surface area and roughness. A density of six molecules per nm^2^ was sufficient to produce monodisperse silica cubes based on a hematite surface area determined through krypton sorption measurements (S_BET_ = 3.7 m^2^ g^−1^), and PVP with a molecular weight of 40 kg mol^−1^. Following physisorption of PVP, it is important to thoroughly wash the hematite particles in order to remove excess PVP, which can act as a nucleation site and cause the formation of secondary silica particles during the deposition of the silica shell.

Extraction of the hematite core is efficient, and can be completed within 16 h if performed prior to the introduction of the defined mesopores. This indicates that a porous silica shell was obtained. The comparatively low surface area of the hollow silica cubes (S_BET_ = 7.0 m^2^ g^−1^) points to the presence of macropores. The extraction of hematite coincided with a particle color change from red to colorless, and was verified by EDS (Figure S1). SEM images of the extracted particles confirmed that the majority of the particles were intact after extraction, with only small amounts of fractured silica shells being visible.

### 3.3. Pseudomorphic Transformation

**Figure 2:**
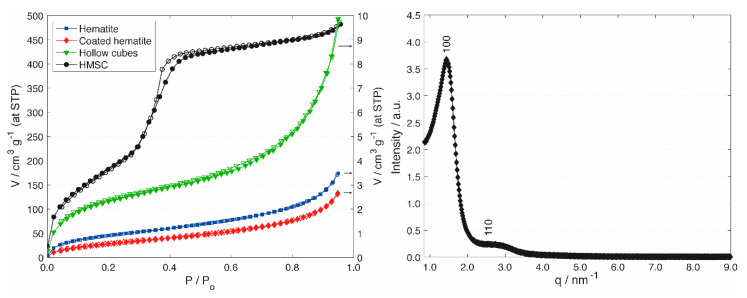
**Left**: Argon sorption isotherms of hematite cubes (blue squares), silica-coated hematite cubes (red diamonds), hollow silica cubes (green triangles) and HMSCs (prepared with CTAB, black circles). Open points denote desorption. **Right**: SAXS pattern of HMSCs (upper limit of PT with CTAB).

Information regarding the development of porosity during the course of the synthesis was obtained by argon sorption measurements (Figure 2). Argon was used to accurately measure the low specific surface area of the silica shells before transformation. The hematite particles featured a type II isotherm with low specific surface area and low pore volume, which is indicative of non-porous or macroporous particles [39]. Upon deposition of the silica shell, the isotherm and specific surface area did not change significantly. After hematite extraction, the specific surface area increased as a result of the loss of the dense hematite core and the accessibility to the interior surface of the silica shells. While these changes were subtle, PT led to a significant change regarding porosity and specific surface area. A type IV isotherm with a distinct pore condensation step was observed after PT, confirming the transformation of the macroporous silica shell to a mesoporous shell with a well-defined pore diameter. The specific surface area increased by a factor of 93, accompanied by a considerable increase in mesopore volume (Table 1). The introduction of ordered mesopores was confirmed through small angle X-ray scattering (SAXS) measurements (Figure 2, Right). The SAXS pattern shows two peaks, which can be indexed on a 2D hexagonal lattice. From the position of the first peak (d_100_), the repeat distance was calculated (a_0_ = 2d_100_/√3 = 5.0 nm). Taking into account the average pore diameter determined by argon sorption (3.8 nm), this results in a pore wall thickness of 1.2 nm.

**Figure 3:**
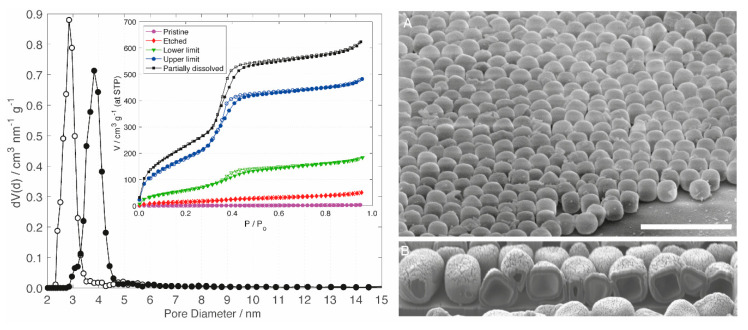
**Left**: Pore size distributions of HMSCs prepared with DTAB (open circles) and CTAB (filled circles). The inset shows argon sorption isotherms of hollow silica cubes after various degrees of PT with CTAB. From bottom to top: Macroporous shell (pristine sample, purple circles), etched shell (red diamonds), lower limit of PT (green triangles), upper limit of PT (blue circles) and partially dissolved shell (black squares). Open points denote desorption. **Right**: SEM image of a HMSC monolayer at a 60° tilt angle (**A**, scale bar = 5 µm) and FIB-SEM image displaying purposely cut Pt-coated HMSCs (**B**).

PT offers the potential to customize the pore size of the silica shell through the use of differently sized SDAs. Dodecyl- and hexadecyltrimethylammonium SDAs led to average pore diameters of 2.8 nm and 3.8 nm, respectively (Figure 3). The degree of PT is increased with increasing NaOH concentration up to a limiting value, above which the dissolution of the silica shell becomes apparent. A low degree of transformation is obtained under mild basic conditions. Transformation is expected to occur on the most accessible surface sites first, which is generally in an inwards direction [30,32]. Under such mild conditions, etching of the external surface and the pore entrances is prevalent. This is indicated by the slightly increased specific surface area and the lack of primary mesopore volume (Table 1). As the degree of PT is increased, the shape of the adsorption isotherm transitions from a type II to a type IV (Figure 3). The development of a defined pore condensation step is accompanied by an increase of the slope in the low relative pressure range and a concomitant increase of the specific surface area. The upper limit of PT implies the introduction of a mesoporous structure with a defined pore size, yielding a large primary mesopore volume without affecting the cubic particle morphology. Still apparent is a small hysteresis at the knee of the desorption branch, which is indicative of cavitation for a small number of macropores on the interior, with respect to the newly generated mesopores [40]. Further increase of the NaOH concentration beyond this upper limit led to the partial dissolution of the silica shell, which manifested itself through a substantial amount of non-cubic secondary silica particles (Figure S3).

### 3.4. Shell Thickness

The thickness of the silica shell can be determined by observing fractured particles in the SEM images. However, this method does not necessarily provide a representative value for the shell thickness, as particles with thinner shells might be more susceptible to fracturing.

The close-packed monolayers of the HMSCs were obtained by dispersing the particles in H_2_O, pipetting the resulting suspension onto a plane substrate and drying it at room temperature (Figure S4). Multilayers can be obtained by the same convenient drop-casting procedure. Such multilayers of HMSCs on Si wafers were found to be suitable for focused ion beam milling after coating with a 20 nm-thick layer of platinum. The HMSC layers were sufficiently stable to allow for the generation of trenches (Figure S5) and the exposure of the internal cavity (Figure 3, Right). From these FIB-SEM images, an average silica shell thickness of approximately 100 nm was calculated.

## 4. Conclusions

We have reported a method to produce hollow mesoporous silica cubes (HMSCs) by means of silica shell formation on a hard template, core extraction and pseudomorphic transformation. The resulting HMSCs feature a large cavity volume (approx. 1.12 cm^3^ g^−1^), large specific surface area and ordered mesopores, yielding a combined void volume of 1.73 cm^3^ g^−1^. The cubic morphology allows for the convenient preparation of close-packed monolayers. Mass transport across the silica shell is strongly affected by the presence of mesopores. Materials featuring a large void volume accessible via such defined mesopores can act as a versatile platform for research in the field of drug delivery and catalysis [2,41], as well as allowing for novel synthetic strategies based on a ship-in-a-bottle approach [42,43,44,45].

## Figures and Tables

**Table 1:** Total specific surface area (S_BET_), total pore volume (V_tot_) and primary mesopore volume (V_p_) of hollow silica cubes after various degrees of PT. V_tot_ includes pores smaller than ca. 40 nm, whereas V_p_ is a measure for the contribution of mesopores between 2 and 8 nm (Figure S2). The data were calculated from argon adsorption isotherms.

	m (NaOH)/mg	S_BET_/m^2^ g^−1^	V_tot_/cm^3^ g^−1^	V_p_/cm^3^ g^−1^
Macroporous shell (pristine)	—	7	0.01	0.00
Etched shell	1.2	52	0.06	0.04
Lower limit of PT	6.4	221	0.23	0.19
Upper limit of PT	9.0	651	0.61	0.53
Partially dissolved shell	12.0	801	0.79	0.68

## Supplementary Materials

The following are available online at https://www.mdpi.com/1996-1944/13/11/2474/s1: Figure S1. Energy-dispersive X-ray spectra. Edge of silica-coated hematite (A), centre of silica-coated hematite (B), edge of hollow silica cube (C), centre of hollow silica cube (D). Scale bars are 1 µm, Figure S2. Contribution of primary mesopores to the pore volume after pseudomorphic transformation under different conditions. From bottom to top: Macroporous shell (pristine), etched shell, lower limit of transformation, upper limit of transformation, partially dissolved shell, Figure S3. SEM images of hollow silica cubes after pseudomorphic transformation under conditions corresponding to the upper limit (A) and under conditions leading to partial dissolution (B). Scale bars are 10 µm, Figure S4. SEM image of a monolayer of HMSCs. The scale bar is 10 µm, Figure S5. SEM images of focused ion beam milled HMSCs. The inset illustrates different degrees of particle sectioning. The scale bars are 5 µm and 1 µm (inset).
