# Supplementary material for: Hollow Silica Cubes with Customizable Porosity
Source: Materials (Basel). 2020 May 29;13(11):2474. doi: 10.3390/ma13112474 (PMC7321225; doi:10.3390/ma13112474)
Supplement: Supplementary file 1 [file materials-13-02474-s001.pdf]

materials

MDPI

Supplementary Information

Hollow Silica Cubes with Customizable Porosity

Samuel Hugh Gallagher, Olivier Trussardi, Oliver Lipp and Dominik Brühwiler *

Institute of Chemistry and Biotechnology, Zurich University of Applied Sciences (ZHAW), CH-8820 Wädenswil, Switzerland; samuel.gallagher@zhaw.ch (S.H.G.); otrussardi@hotmail.com (O.T.); oliver.lipp@zhaw.ch (O.L.)

* Correspondence: dominik.bruehwiler@zhaw.ch

1. Extraction of the Hematite Core

EDS confirms the successful extraction of the hematite core after coating with silica (Figure S1). No Fe signals were observed for the hollow silica cubes. The carbon signals are due to the carbon tape used for mounting the samples.

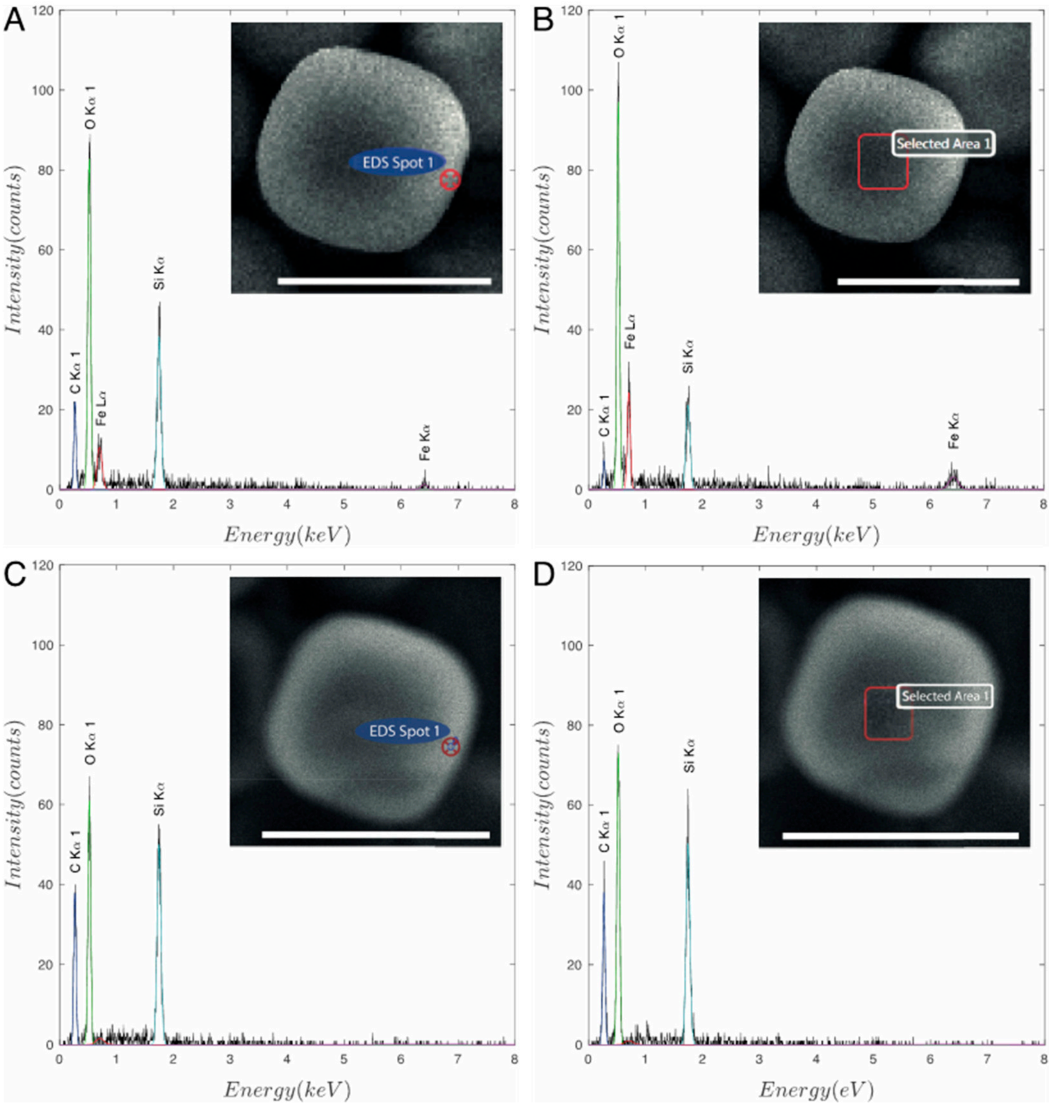

Figure S1. Energy-dispersive X-ray spectra. Edge of silica-coated hematite (**A**), centre of silica-coated hematite (**B**), edge of hollow silica cube (**C**), centre of hollow silica cube (**D**). Scale bars are 1 µm.

2. Pseudomorphic Transformation

The conditions of the pseudomorphic transformation (PT) must be carefully adjusted to obtain a maximum primary mesopore volume without degradation of the particle morphology, i.e., without dissolution of the silica shell. Figure S2 shows the development of the primary mesopore volume under increasingly harsh transformation conditions using CTAB as SDA. The following amounts of NaOH were used: 1.2 mg (etched shell), 6.4 mg (lower limit of transformation), 9.0 mg (upper limit of transformation) and 12.0 mg (partially dissolved shell).

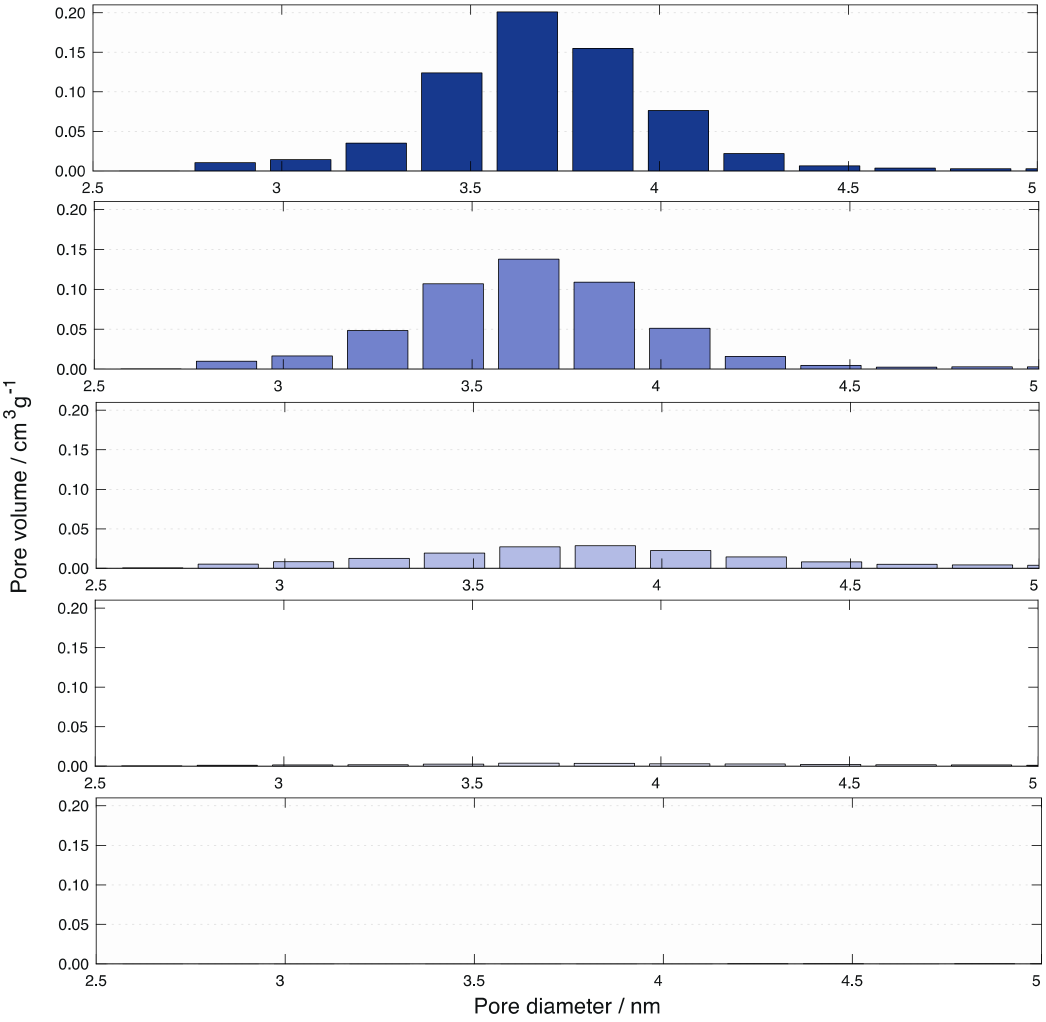

Figure S2. Contribution of primary mesopores to the pore volume after pseudomorphic transformation under different conditions. From bottom to top: Macroporous shell (pristine), etched shell, lower limit of transformation, upper limit of transformation, partially dissolved shell.

The upper limit of transformation implies the introduction of a mesoporous structure with a defined pore size, yielding a large primary mesopore volume without affecting the particle morphology. Figure S3A shows the SEM image of a sample that has undergone PT (upper limit). The cubic morphology is well preserved. At higher NaOH concentration, partial dissolution of the hollow silica cubes occurs, leading to the formation of secondary particles and agglomerates (Figure S3B). It should be mentioned that the resolution of the scanning electron microscope (Quanta FEG 250) is insufficient for reliable imaging of pores with a diameter below 5 nm.

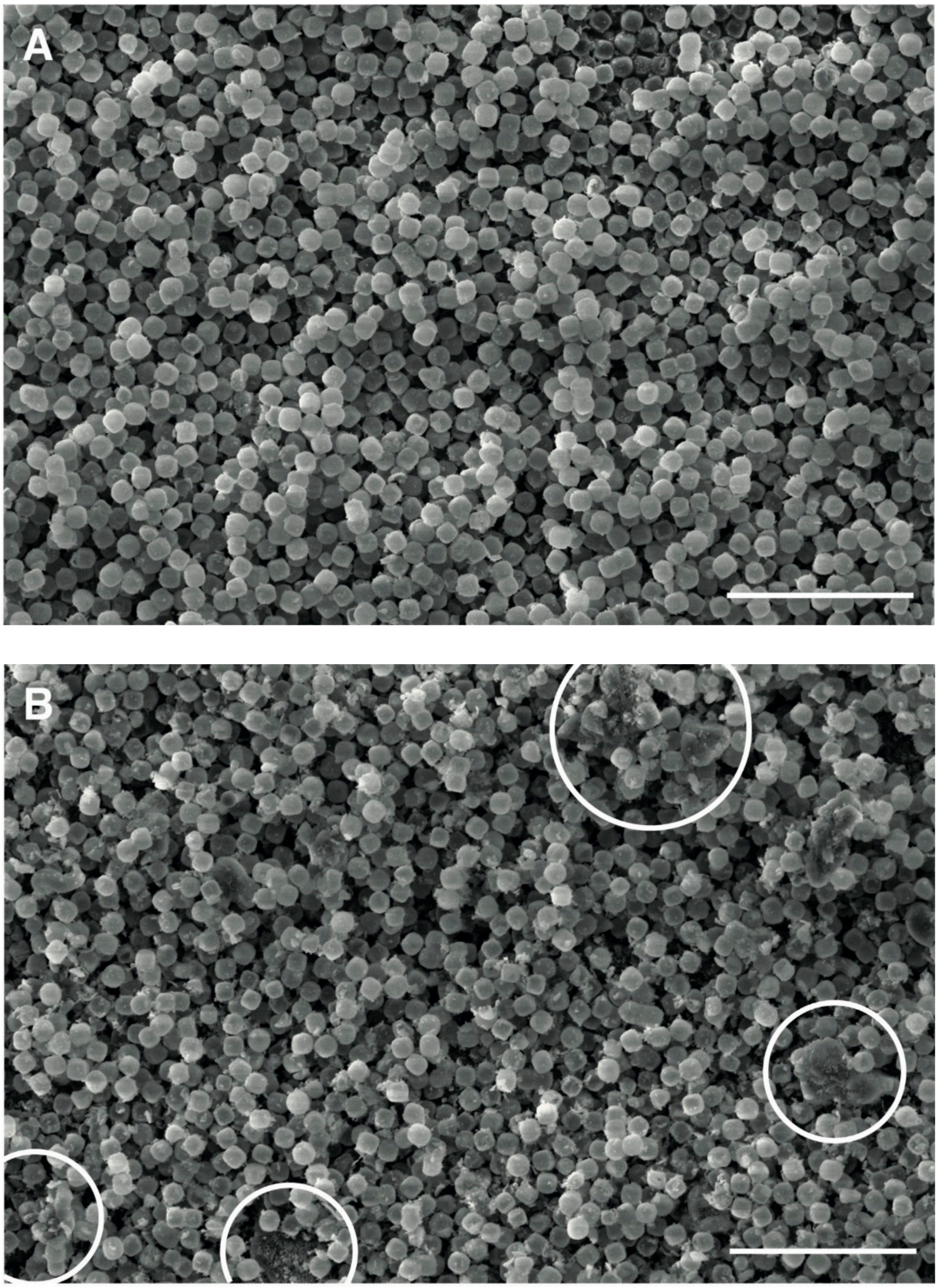

Figure S3. SEM images of hollow silica cubes after pseudomorphic transformation under conditions corresponding to the upper limit (**A**) and under conditions leading to partial dissolution (**B**). Scale bars are 10 μm.

3. Formation of Monolayers and Focused Ion Beam Milling

Arrangement of the hollow mesoporous silica cubes (HMSCs) into monolayers was achieved by means of a simple drop casting method. SEM images show dense packing of the HMSCs (Figure S4). The large cavity of the HMSCs was imaged by focused ion beam milling combined with SEM (FIB-SEM). Figure S5 shows that the HMSC multilayers are sufficiently stable to allow for the generation of trenches.

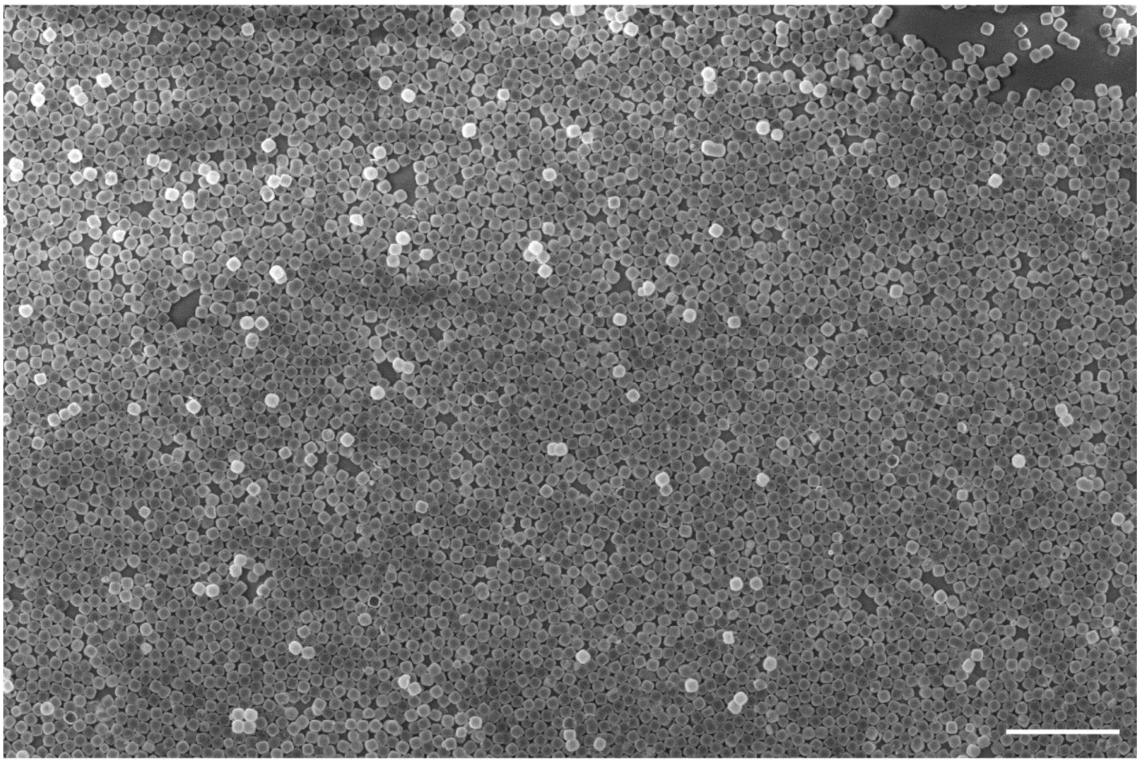

Figure S4. SEM image of a monolayer of HMSCs. The scale bar is 10 µm.

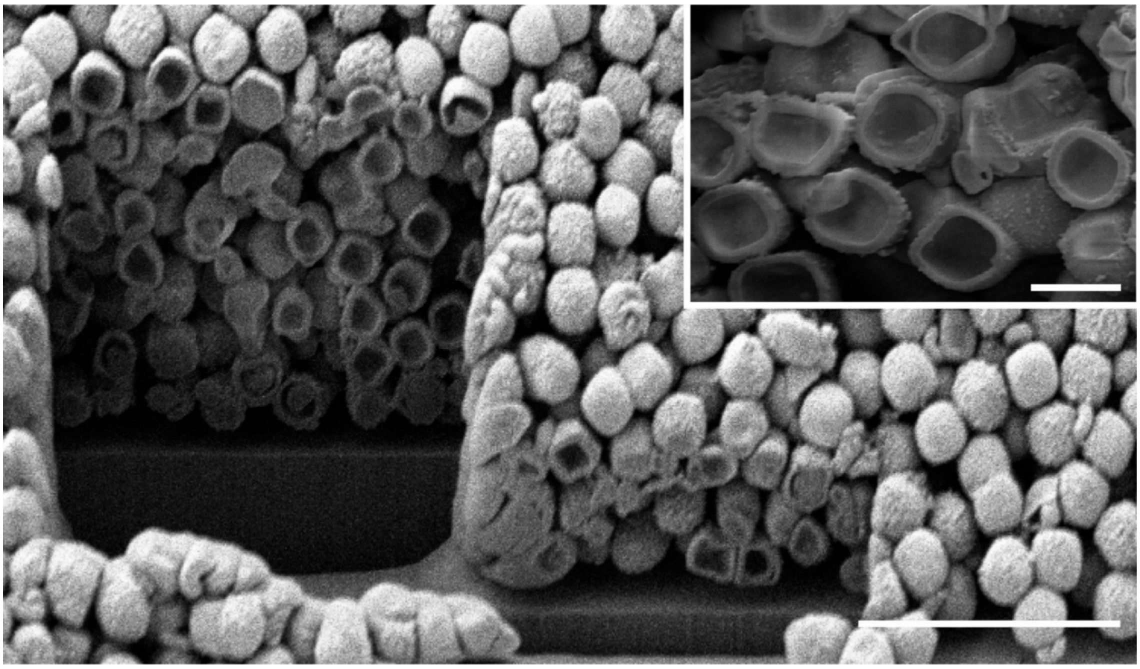

Figure S5. SEM images of focused ion beam milled HMSCs. The inset illustrates different degrees of particle sectioning. The scale bars are 5 µm and 1 µm (inset).
